# Essential Skills Development Needs of High School Students in Southern Thailand for Work in the 21st Century Labour Market

**DOI:** 10.1192/j.eurpsy.2023.938

**Published:** 2023-07-19

**Authors:** K. Janyam

**Affiliations:** Faculty of Liberal Arts, PRINCE OF SONGKLA UNIVERSITY, Songkhla, Thailand

## Abstract

**Introduction:**

Presently, the concept of preparing youth for the labor market in the 21st century receives much attention because of the rapidly changing nature of work and the soft skills and hard skills required by employers. Therefore, graduates need to be competent at work skills necessary for the 21st century to be able to face challenges of work in the present age and to have new perspectives for facing challenges and changes of work. According to the Federation of Thai Industries, the estimated number of workers needed by 14 groups of manufacturing industries during 2013-2017 included 58 percent of high school graduates.

**Objectives:**

The current study aims to survey and assess essential skills development in high school students in Southern Thailand for the workforce in the 21st century.

**Methods:**

The cross-sectional study was applied to 1,200 subjects consisting of 400 employers and 800 high school students. Data was analysed using means, standard deviation and t-test

**Results:**

The results revealed that communication, digital literacy, creativity, critical thinking, collaboration, and responsibility were the major skills development needs of high school students in the 21st century labor market. The results indicated that high school students’ levels of skills were lower than those required by employers in four skills: communication, digital literacy, critical thinking, and creativity at the statistical significant level of .001, as presented in table 1.

**Table 1.** Means different for skills development need by sample group.

**Skills**	**Employers**	**High school students**			
	Χ	**S.D.**	χ	**S.D.**	**t**
Communication	4.08	.40	3.17	.89	21.385***
Digital literacy	4.17	.41	3.41	.97	17.120***
Creativity	3.97	.45	3.01	.79	17.113***
Critical thinking	4.14	.43	3.47	.97	14.776***
Collaboration	3.39	1.04	3.41	.94	-4.267***
Responsibility	3.39	1.18	3.61	1.00	-3.877***

P<.001

**Conclusions:**

The high school students possess skills needs for work in the 21st century at lower levels than those needed by employers. This is the issue requiring attention from individuals involved in educational management as they must find ways to develop the teaching and learning methods that can upgrade students’ skills that are needed for work and seen by employers as highly important. These skills are communication, digital literacy, critical thinking, and creativity. The teaching and learning styles should be more active rather than passive to stimulate students to think, to be proactive instead of passively to respond to whatever happens, to propose their ideas or to express differing opinions and using the appropriate language or communicating tools. The fact that these skills are at a moderate level could result in limitations for young people’s development or employment as these skills are required for work in this century where analytical thinking and creativity are necessary for invention both in the workplace and in private life.

**Disclosure of Interest:**

None Declared

